# Low-Complexity Noncoherent Demodulation Method for Underwater Electromagnetic Communication

**DOI:** 10.3390/s26072266

**Published:** 2026-04-07

**Authors:** Longyang Deng, Deguang Zhao, Bizheng Liang, Xuhui Ding, Ziyi Yang, Dekang Liu

**Affiliations:** 1 School of Cyberspace Science and Technology, Beijing Institute of Technology, Beijing 100081, China; 2 Institute of Telecommunication and Navigation Satellites, China Academy of Space Technology, Beijing 100094, China; 3 Advanced Technology Research Institute, Beijing Institute of Technology, Jinan 250098, China; 4 Institute of Unmanned Systems, Beihang University, Beijing 100191, China

**Keywords:** MSK, noncoherent demodulation, maximum likelihood, resource reuse, adaptive extension, underwater electromagnetic communication

## Abstract

To strike a balance between complexity and performance in Minimum Shift Keying (MSK) systems for underwater electromagnetic communication, we propose a low-complexity maximum-likelihood (ML) noncoherent demodulation method. By integrating a resource reuse mechanism with a confidence-driven adaptive extension strategy, the proposed method significantly reduces computational resource consumption while maintaining near-optimal demodulation performance. Simulation results demonstrate that the bit-error-rate (BER) performance of the proposed method approaches that of the traditional fixed length ML receiver when the confidence threshold is set to 0.1. Meanwhile, the proposed method reduces complex correlation operations by 96.2% and complex addition operations by 87.1%, achieving minimal average computational overhead. Furthermore, we evaluate the method under frequency-flat Rayleigh fading channels, and the results confirm that the proposed method retains its performance advantage and complexity reduction under fading, supporting its potential for reliable underwater communication.

## 1. Introduction

Underwater wireless communication is essential for ocean exploration and autonomous underwater vehicle (AUV) operations [1,2]. While underwater acoustic communication (UWAC) is the most established for long-range links, its performance is highly dependent on channel geometry [3]. Vertical channels typically suffer from depth-dependent sound speed profiles, whereas shallow-water horizontal channels face severe time-varying multipath interference due to surface-bottom reflections and refractive properties [4]. Furthermore, UWA is constrained by low propagation speeds (1500 m/s), high latency, and an inability to cross the air–water interface [5].

**Figure 1:**
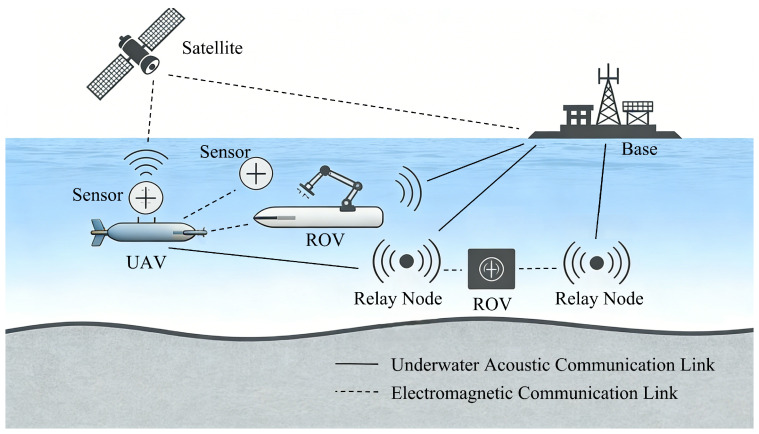
Underwater communication scenario.

In contrast, underwater electromagnetic communication (UEMC) provides higher bandwidth, lower latency, and better robustness against acoustic noise and turbidity [6]. As re-evaluated in [6], RF signals are particularly effective for short-range underwater sensor networks (UWSNs) due to their capability for cross-boundary communication. Nevertheless, the conductive nature of seawater (typically σ≈4 S/m) presents significant challenges [7]. Electromagnetic waves undergo substantial absorption, where attenuation increases exponentially with frequency [8]. This dispersive characteristic leads to frequency-selective fading, which is further exacerbated by interface effects such as lateral waves and Goos–Hänchen (GH) shifts at the sea-air/sea-floor boundaries [9]. Figure 1 shows the typical underwater communication scenario.

To ensure reliable data transmission under these harsh conditions, the choice of modulation is critical. In UWAC, orthogonal frequency-division multiplexing (OFDM) has been widely adopted to combat multipath interference [10], while M-ary chirp spread spectrum (CSS) offers robustness against Doppler dispersion [11]. For UEMC, two complementary modulation strategies have emerged to address the channel’s frequency-dependent attenuation and limited bandwidth. Phase-coherent schemes such as BPSK and QPSK concentrate energy in narrow bandwidths, offering resilience to frequency-selective fading and Doppler shifts [12]. Constant-envelope schemes such as FSK encode information in frequency rather than phase, providing inherent tolerance to random phase and amplitude fluctuations; this makes FSK particularly effective in the extremely low-frequency (ELF) band for long-range links [13].

MSK uniquely combines the advantages of both approaches. Its constant envelope property allows for the use of high-efficiency non-linear power amplifiers without significant spectral regrowth [14], which is vital for extending the battery life of submerged sensors. Simultaneously, its continuous phase and compact spectrum improve the spectral efficiency, offering improved performance in bandlimited channels [15]. These attributes position MSK as an attractive choice for UEMC.

Despite its advantages, the implementation of MSK receivers faces a trade-off between performance and complexity. Coherent demodulation is often impractical in dynamic underwater links due to the difficulty of carrier synchronization [16,17]. Noncoherent demodulation offers better robustness but typically results in a performance penalty [18].

Specifically, noncoherent techniques for MSK can be broadly categorized into two main distinct approaches: single-symbol detection and multi-symbol detection. Single-symbol detection makes decisions on each received symbol using only local information [19], among which one-bit differential detection is the most representative due to its low complexity [20]. However, such single-symbol differential detection suffers from a performance loss of about 3–4 dB compared to coherent detection [21].

In contrast, multi-symbol detection exploits a block of received samples over an observation window and performs a joint decision that captures inter-symbol relationships [22,23]. A practical family within this category is multi-symbol differential detection, which improves performance by observing multiple symbols [24] and can be further enhanced via decision feedback [25] or post-processing [26]. However, these differential detectors fundamentally approximate the likelihood metric and, thus, incur irreducible performance penalties, particularly under fast fading [27].

A systematic ML noncoherent framework based on direct likelihood computation was first established by Osborne and Luntz for CPFSK, showing that performance approaches coherent detection as the window increases, but at the cost of exponential complexity [28]. Leib and Pasupathy extended this framework to MSK, revealing that traditional differential and envelope detectors are special cases of the ML receiver, and further demonstrated that exploiting MSK’s phase continuity enables reduced-complexity estimation with minimal performance loss [29]. Despite these advances, the performance–complexity trade-off remains a key challenge for practical noncoherent MSK reception, especially when long observation windows are required.

Existing research primarily reduces the complexity of ML noncoherent demodulation techniques along two directions: metric approximation and search control. The first direction approximates or simplifies the ML criterion, such as using Bessel function approximation [28] or simplifying the phase function [24,29]. The second direction reduces the number of candidate paths through sequence search strategies, such as employing decision feedback [25,27] or differential demodulation-based pre-decision [30]. However, these approaches often introduce non-negligible performance degradation alongside reduced complexity, failing to resolve the trade-off between performance and computational complexity.

Building upon reduced-complexity noncoherent MSK receivers, this work can be viewed as a further development along both the metric-approximation and the search-control directions. On the metric side, under the standard Bessel-based noncoherent ML formulation, we exploit the decomposable MSK phase structure to enable correlation resource reuse: Repeated correlations across candidate sequences are converted into a small set of reusable base integrals with lightweight sign operations, thereby substantially reducing the dominant correlation workload and decoupling the oversampling factor from the exponential search term. On the search-control side, we introduce a confidence-driven adaptive window-extension strategy, where the confidence metric quantifies decision reliability and triggers extension only when necessary. This regulates the effective observation window and reduces the average search effort while preserving near-ML performance.

The main contributions are as follows:

1. We develop a likelihood computation mechanism based on a correlated resource reuse mechanism. By analyzing the decomposable phase structure of MSK signals, the exponentially correlated complex correlation in the traditional ML method is transformed into a linear number of pre-computed and reusable base integral terms. This significantly reduces the exponential growth coefficient of the arithmetic overhead. (Note that the proposed method alleviates the dominant correlation-cost bottleneck but does not eliminate the exponential dependence due to exhaustive sequence search).
2. We provide a confidence-driven, adaptive observation-window extension strategy. A quantifiable confidence metric for decision reliability is constructed. Based on this metric, the observation window is extended adaptively, enabling on-demand allocation of demodulation resources. This minimizes the average computational overhead while ensuring overall performance.
3. We provide a two-tiered validation of the proposed scheme. Its baseline performance is first established in an attenuation-dominated AWGN channel, followed by a robustness analysis under frequency-flat Rayleigh fading to simulate challenging multipath environments. The results confirm its applicability across diverse underwater scenarios.

The structure of this paper is organized as follows: Section 2 establishes the system model and briefly describes traditional ML noncoherent demodulation. Section 3 elaborates in detail on the proposed resource reuse mechanism and the confidence-based extension strategy. Section 4 validates the performance and efficiency of the proposed method through simulation experiments. This paper is concluded in Section 5.

## 2. System Model

**Figure 2:**
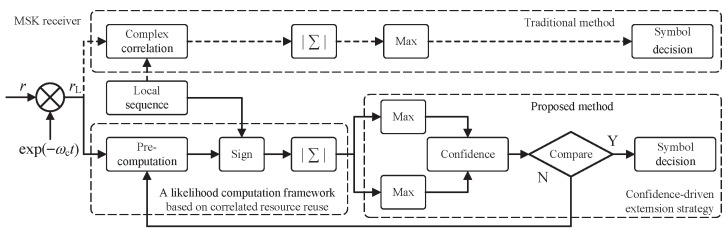
MSK noncoherent demodulation system based on ML principle.

In this section, we consider an MSK noncoherent demodulation system based on the ML principle, as depicted in Figure 2. To accurately characterize the underwater electromagnetic environment, we establish a signal model that accounts for both deterministic path loss and stochastic multipath fading.

### 2.1. Signal Model

The transmitted MSK signal can be expressed as

(1) $$s\left(t\right)=\cos\left[2\pi f_{c}t+\varphi \left(t;a_{1}^{n+m}\right)\right],\;0\leq t\leq \left(n+m\right)T,$$

where $f_{c}$ is the carrier frequency, $a_{1}^{n+m}$ is the the transmission sequence, and *T* is the symbol period. The transmission sequence $a_{1}^{n+m}$ is composed by the binary symbol $a_{i}\in \left\{\pm 1\right\}$ and can be expressed as $\left[a_{1},a_{2},\ldots ,a_{n+m}\right]$, where *m* is a positive integer less than *n*.

The phase trajectory $\varphi \left(t;a_{1}^{n+m}\right)$ is shaped by the transmitted sequence and can be expressed as

(2) $$\varphi \left(t;a_{1}^{n+m}\right)=\pi \sum_{i=1}^{n+m}a_{i}q\left[t-\left(i-1\right)T\right],$$

where $q\left(t\right)$ is the linear phase pulse and is defined as

(3) $$q\left(t\right)=\left\{\begin{array}{cc} 0, & t<0\\ t/(2T), & 0\leq t\leq T\\ 1/2, & t>T \end{array}\right..$$

### 2.2. Underwater Electromagnetic Channel Model

In underwater electromagnetic communication, the received signal is affected by both deterministic propagation attenuation and stochastic environmental fluctuations. The complex baseband equivalent of the received signal can be represented as

(4) $$r_{L}\left(t;a_{1}^{n+m},\theta \right)=h\left(t\right)\cdot \exp j\left[\varphi \left(t;a_{1}^{n+m}\right)+\theta \right]+n_{L}\left(t\right),\;0\leq t\leq \left(n+m\right)T,$$

where $n_{L}\left(t\right)$ is complex additive white Gaussian noise; θ represents the unknown constant carrier phase offset. The channel coefficient $h\left(t\right)$ represents the combined effects of propagation loss and multi-path fading.

Depending on the communication range and environmental geometry, two primary channel regimes are considered in this work:

1. Attenuation-Dominated Regime (AWGN): In short-range scenarios, the direct path between the transmitter and receiver is significantly stronger than any reflected components. In this case, h(t) can be treated as a constant factor representing exponential path loss. Thus, the channel is simplified to an AWGN model, which is a common assumption for evaluating the baseline performance of UEMC systems [8].
2. Fading-Dominated Regime (Rayleigh Fading): As the distance increases or when surface/bottom reflections become non-negligible, the receiver collects multiple signal components with random phases [9]. In such cases, h(t) is modeled as a stochastic process following a Rayleigh distribution:
  (5) $$p\left(|h|\right)=\frac{\left|h\right|}{\sigma^{2}}e^{-\frac{{\left|h\right|}^{2}}{2\sigma^{2}}},\;\left|h\right|\geq 0,$$
  where $\sigma^{2}$ is the variance of the multipath components; |h| is the envelope of the channel coefficient $h\left(t\right)$. To simulate the temporal correlation of the channel, we adopt the Jakes’ Doppler spectrum, where the time-varying nature of h(t) is determined by the maximum Doppler shift $f_{d}$.

### 2.3. Noncoherent Metric and Observation Window

To demodulate the *n*-th symbol $a_{n}$, the traditional ML method requires traversing all local sequences within a symmetric observation window centered around *n*. Let the *k*-th local sequence be $b_{n-m}^{n+m}\left(k\right)=\left[b_{n-m}\left(k\right),\ldots ,b_{n}\left(k\right),\ldots ,b_{n+m}\left(k\right)\right]$, where $b_{i}\in \left\{\pm 1\right\}$ is the *i*-th local symbol. With a window length of N=2m+1, the traversal count is $2^{N}$.

For each local sequence, the likelihood is computed through complex correlation and coherent accumulation based on the received signal and local signal. Therefore, the *k*-th likelihood Λ(k,N) can be expressed as

(6) $$\Lambda \left(k,N\right)=\left|\sum_{j=n-m}^{n+m}\int_{(j-1)T}^{jT}r_{L}\left(t;a_{1}^{n+m},\theta \right)\cdot \exp j\left\{-\varphi \left[t;b_{1}^{n+m}\left(k\right)\right]\right\}dt\right|,$$

where $\varphi \left[t;b_{1}^{n+m}\left(k\right)\right]$ is the phase trajectory of local signal.

By comparing the likelihoods of all sequences, the sequence with the maximum likelihood is selected, and the corresponding central symbol is output as the demodulation result. Let the index of the optimal sequence be $\hat{k}$, then the demodulation result can be expressed as

(7) $${\hat{a}}_{n}=b_{n}\left(\hat{k}\right),\;\hat{k}=\mathrm{argmax}\Lambda \left(k,N\right).$$

It should be noted that the noncoherent metric Λ(k,N) in Equation (6) is fundamentally derived under the premise of an unknown but constant phase over the observation window. When applied to a time-varying fading channel, the effectiveness of this metric heavily relies on the assumption that the channel fading coefficient h(t) varies slowly (i.e., remains quasi-static) within the window length. This holds when the window duration NT is significantly shorter than the channel coherence time $T_{c}$. When the Doppler spread increases such that NT becomes comparable to $T_{c}$, the metric becomes mismatched and an error floor may appear. This physical constraint naturally limits the maximum effective window length and motivates the confidence-driven adaptive window extension strategy in Section 3.2, which explicitly bounds the window growth to balance robustness and complexity.

## 3. A Low-Complexity MSK ML Demodulation Method

As shown in Equation (6), the traditional ML noncoherent method faces a critical dilemma in practical underwater electromagnetic (EM) applications. On one hand, its computational complexity grows exponentially with the observation window length *N*, forcing systems to adopt extremely small windows (typically N=3) that limit demodulation performance under severe AWGN. On the other hand, as analyzed in Section 2.3, simply enforcing a fixed, lengthy observation window is not a universal solution. In fading-dominated regimes, an overly long window may exceed the channel’s coherence time, leading to phase cancellation and catastrophic likelihood distortion.

To fundamentally resolve this trade-off between computational complexity, noise suppression, and phase stability, we propose a two-stage low-complexity demodulation scheme. This scheme combines a structural resource reuse mechanism with a confidence-driven adaptive strategy.

### 3.1. Likelihood Calculation Method Based on Correlated Resource Reuse

The core redundancy of the traditional method lies in repeatedly computing essentially identical integral operations for each local sequence. To reduce this redundancy, we design an efficient pre-computation and reuse mechanism based on the decomposable nature of the MSK signal phase structure.

Consider the local signal within a symbol period. During the *j*-th symbol period, the local signal phase can be decomposed into the sum of a linear time-varying term and a constant phase term:

(8) $$\varphi \left[t;b_{1}^{n+m}\left(k\right)\right]=\frac{\pi b_{j}\left(k\right)}{2T}t+\frac{\pi }{2}c_{j}\left(k\right),\;\left(j-1\right)T\leq t\leq jT,$$

where $c_{j}\left(k\right)$ is the cumulative phase state determined by the local sequence and can be expressed as

(9) $$c_{j}\left(k\right)=\sum_{i=1}^{j}\left[b_{i}\left(k\right)-b_{j}\left(k\right)\right]\;\;\mathrm{mod}\;4.$$

At this point, the complex correlation in the likelihood calculation can be expressed as:

(10) $$\begin{array}{cc} x_{j}\left[a_{1}^{n+m},\theta ,b_{1}^{n+m}\left(k\right)\right] & =\int_{(j-1)T}^{jT}r_{L}\left(t;a_{1}^{n+m},\theta \right)\cdot \exp j\left\{-\varphi \left[t;b_{1}^{n+m}\left(k\right)\right]\right\}dt\\ \; & =\exp j\left[-\frac{\pi }{2}c_{j}\left(k\right)\right]\cdot \int_{(j-1)T}^{jT}r_{L}\left(t;a_{1}^{n+m},\theta \right)\exp j\left[-\frac{\pi b_{j}\left(k\right)}{2T}t\right]dt. \end{array}$$

Equation (10) indicates that the complex correlation can be decomposed into the product of an integral term and a phase rotation term.

The value of the integral term depends solely on the current symbol $b_{j}\left(k\right)$, and is independent of other symbols in the sequence. Therefore, for an observation window of length *N*, there are only 2N fundamentally distinct basic integral terms. The complex correlations of all sequences are obtained by applying different phase rotations to these 2N basic integral terms.

Furthermore, the cumulative phase state $c_{j}\left(k\right)$ controlling the phase rotation term is analyzed. According to Equation (9), $c_{1}\left(k\right)=0$; when j≠1, the recursive form of $c_{j}\left(k\right)$ can be expressed as

(11) $$c_{j}\left(k\right)=\left\{\begin{array}{ccc} c_{j-1}\left(k\right), & \; & b_{j-1}\left(k\right)=b_{j}\left(k\right)\\ c_{j-1}\left(k\right)+2\left(j-1\right)b_{j-1}\left(k\right) & \;\mathrm{mod}\;4 & b_{j-1}\left(k\right)\neq b_{j}\left(k\right) \end{array}\right..$$

Given the initial condition $c_{1}\left(k\right)=0$, the recursive form indicates that $c_{j}\left(k\right)$ is confined to the set $\left\{0,2\right\}$, meaning the phase rotation term is an integer multiple of π. Thus, the complex multiplication corresponding to phase rotation can be simplified to a sign-flipping operation:

(12) $$\exp j\left[-\frac{\pi }{2}c_{j}\left(k\right)\right]=\left\{\begin{array}{cc} 1, & c_{j}\left(k\right)=0\\ -1, & c_{j}\left(k\right)=2 \end{array}\right.=1-c_{j}\left(k\right).$$

**Figure 3:**
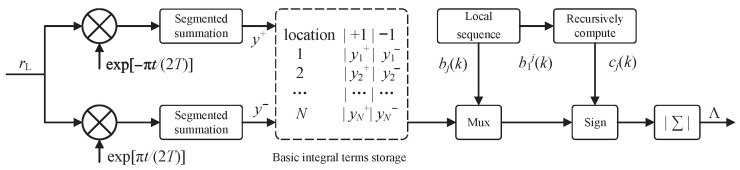
Likelihood calculation method based on correlated resource reuse.

Based on the above analysis, we propose a likelihood calculation method based on correlated resource reuse. Its flow is shown in Figure 3, with specific steps as follows:

First, pre-compute basic integral terms. By down-converting the complex baseband received signal and performing segmented summation, two types of basic integral terms corresponding to $b_{j}\left(k\right)=+1$ and $b_{j}\left(k\right)=-1$ can be obtained:

(13) $$\begin{array}{cc} \; & y_{j}^{+}=\int_{(j-1)T}^{jT}r_{L}\left(t;a_{1}^{n+m},\theta \right)\exp j\left(-\frac{\pi }{2T}t\right)dt\\ \; & y_{j}^{-}=\int_{(j-1)T}^{jT}r_{L}\left(t;a_{1}^{n+m},\theta \right)\exp j\left(+\frac{\pi }{2T}t\right)dt. \end{array}$$

Store these two types of basic integral terms, totaling 2N terms.

Then, recursively compute the cumulative phase state. Based on the local sequence $b_{1}^{j}\left(k\right)$, the cumulative phase state $c_{j}\left(k\right)$ is recursively computed according to Equation (11).

Finally, compose sequence likelihood. Select the basic integral term from the storage table based on the current symbol $b_{j}\left(k\right)$, apply the sign flip determined by the cumulative phase state $c_{j}\left(k\right)$, and compose the sequence likelihood after accumulation and magnitude operation:

(14) $$\Lambda \left(k,N\right)=\left|\sum_{j=n-m}^{n+m}\left[1-c_{j}\left(k\right)\right]\cdot \left\{y_{j}^{+}\cdot \delta \left[b_{j}\left(k\right)-1\right]+y_{j}^{-}\cdot \delta \left[b_{j}\left(k\right)+1\right]\right\}\right|,$$

where $\delta \left(\cdot \right)$ is the Kronecker delta function.

Within the proposed mechanism, we pre-compute a set of basic correlation values and use lightweight sign flip operations to derive all local sequence correlation values, thereby avoiding the extensive redundant correlation calculations in traditional methods.

Furthermore, this mechanism exhibits an excellent incremental computation property: When the observation window expands from *N* to N+ΔN, only 2ΔN basic integral terms need to be recomputed, with the original results fully reused, providing efficient support for adaptive extension strategies.

### 3.2. Adaptive Observation Window Extension Strategy Based on Confidence

Although the resource reuse mechanism reduces the computational cost of long windows, fixed long windows remain suboptimal. An ideal solution should dynamically evaluate the reliability of short-window decisions, triggering extension only when necessary. To this end, we design a lightweight adaptive strategy based on a confidence metric in this subsection.

Consider the binary hypothesis for the central symbol $a_{n}$:

(15) $$H_{0}:a_{n}=+1\;H_{1}:a_{n}=-1.$$

If all sequences have equal prior probability, the noncoherent likelihood function for hypothesis $H_{i}$ can be expressed as follows [28]:

(16) $$p\left(r_{L}|H_{i}\right)=\sum_{k\in S_{i}}I_{0}\left[\frac{2}{N_{0}}\Lambda \left(k,N\right)\right],\;i=0,1,$$

where $S_{i}$ denotes the index set corresponding to the sequence under the hypothesis $H_{i}$, $I_{0}\left(\cdot \right)$ is the zero-order modified Bessel function, $N_{0}$ is the noise power spectral density, and $\Lambda \left(\cdot \right)$ is the likelihood mentioned earlier.

At a high signal-to-noise ratio ($2\Lambda \left(k,N\right)/N_{0}\gg 1$), the summation term in Equation (16) is dominated by its largest component. Based on this characteristic, the original likelihood function can be approximated as follows using the monotonic increasing property of Bessel function:

(17) $$p\left(r_{L}|H_{i}\right)\approx \max_{k\in S_{i}}I_{0}\left[\frac{2}{N_{0}}\Lambda \left(k,N\right)\right]\propto I_{0}\left[\frac{2}{N_{0}}\max_{k\in S_{i}}\Lambda \left(k,N\right)\right].$$

Furthermore, using the approximation in Equation (17), we construct the generalized likelihood ratio:

(18) $$L=\frac{p\left(r_{L}|H_{0}\right)}{p\left(r_{L}|H_{1}\right)}\approx \frac{I_{0}\left(2X_{0}/N_{0}\right)}{I_{0}\left(2X_{1}/N_{0}\right)},$$

where the notation $X_{0}$ and $X_{1}$ are defined for convenience:

(19) $$X_{0}=\max_{k\in S_{0}}\Lambda \left(k,N\right),\;X_{1}=\max_{k\in S_{1}}\Lambda \left(k,N\right).$$

Equation (18) can be simplified by using the high SNR approximation of the Bessel function: $I_{0}\left(z\right)\approx e^{z}/\sqrt{2\pi z}$. The logarithmic form of the simplified generalized likelihood ratio can be expressed as

(20) $$\ln L=\frac{2}{N_{0}}\left(X_{0}-X_{1}\right)+\frac{1}{2}\ln\left(\frac{X_{1}}{X_{0}}\right).$$

When $X_{0}$ and $X_{1}$ differ significantly, the first term on the right dominates; when the two are close, the second term approaches zero. Therefore, the decision information is primarily determined by the first term on the right.

To eliminate the dependence of the generalized likelihood ratio on the unknown noise power and obtain a normalization metric independent of signal energy, the following confidence metric is further constructed:

(21) $$\alpha_{conf}=\left|\frac{2\left(X_{0}-X_{1}\right)/N_{0}}{2\left(X_{0}+X_{1}\right)/N_{0}}\right|=\frac{\left|X_{0}-X_{1}\right|}{X_{0}+X_{1}}\in \left[0,1\right]$$

This metric approaches 1 when the decision is highly reliable and approaches 0 when uncertainty is high.

The simplified metric $\alpha_{conf}$ facilitates an efficient trade-off between computational complexity and operational robustness. Although the approximations exhibit numerical deviations in low-SNR regimes, this structural characteristic functions as a reliable indicator of decision uncertainty. Specifically, the convergence of correlation magnitudes $X_{0}$ and $X_{1}$ under severe noise leads to a predictable deflation of $\alpha_{conf}$, which effectively triggers the adaptive window extension. By increasing the observation duration, the system leverages the noise averaging effect to accumulate additional signal energy, thereby compensating for the mathematical approximation errors through physical-layer reinforcement [31,32,33].

**Figure 4:**
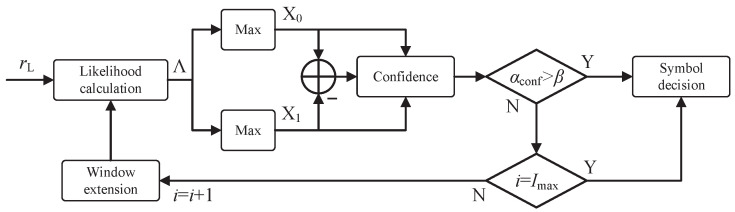
Iterative observation window extension strategy based on confidence.

Based on the confidence metric $\alpha_{conf}$, we design an iterative observation window extension strategy, whose process is illustrated in Figure 4. The strategy presets a confidence threshold β, an extension step size ΔN, and a highly crucial scenario-configurable parameter: the maximum iteration limit $I_{max}$. The execution process proceeds as follows:

First, calculate the likelihood for all sequences based on the base observation window length $N_{b}$. Next, compute the confidence metric $\alpha_{conf}$ and compare it with the threshold β. If $\alpha_{conf}<\beta$, the algorithm initiates an iterative window extension. During each iteration, the observation window is extended by ΔN, and the confidence metric is incrementally re-evaluated. The iteration strictly terminates either when the confidence threshold is met ($\alpha_{conf}\geq \beta$) or when the iteration count reaches $I_{max}$. Consequently, the maximum possible observation window is strictly bounded by $N_{max}=N_{b}+I_{max}\cdot \Delta N$.

Integrating the correlated resource reuse mechanism with this generalized iterative strategy yields a complete low-complexity MSK noncoherent demodulation method, as outlined in Algorithm 1.
**Algorithm 1** A Low-Complexity MSK ML Noncoherent Demodulation1:Initialize $N_{b}$, β, ΔN, and maximum iteration limit $I_{max}$.2:Set current iteration i=0 and current window length $N_{u}=N_{b}$.3:Precompute $y_{j}^{+}$ and $y_{j}^{-}$ using Equation (13).4:**while** True **do**5:   Compute $\Lambda \left(k,N_{u}\right)$ and calculate $\alpha_{conf}$ using Equations (14), (19) and (21).6:   **if** $\alpha_{conf}>\beta$ or $i\geq I_{max}$ **then**7:     Break8:   **else**9:     Increment i=i+1 and update $N_{u}=N_{b}+i\cdot \Delta N$.10:     Incrementally precompute the new basic integral value for the extended segment.11:   **end if**12:**end while**13:Let $\hat{k}=\mathrm{argmax}\Lambda \left(k,N_{u}\right)$ and output symbol decision ${\hat{a}}_{n}=b_{n}\left(\hat{k}\right)$.

From a theoretical and engineering perspective, introducing a finite $I_{max}$ acts as a crucial structural boundary. It ensures that the maximum observation window $N_{max}$ does not exceed the channel’s coherence time $T_{c}$ in time-varying environments, thereby mitigating the risk of destructive phase cancellation. Simultaneously, it strictly bounds the worst-case computational complexity and ensures deterministic processing latency, which is exceptionally vital for energy-constrained underwater nodes. The specific impact of $I_{max}$ and its optimal configuration under varying channel conditions are evaluated in Section 4.

## 4. Simulation and Experimental Results

In this section, we evaluate the proposed method through Monte Carlo simulations. To align with the physical characteristics of underwater EM propagation analyzed in Section 2.2, we adopt a two-tiered validation approach: Baseline Evaluation (AWGN) and Robustness Evaluation (Rayleigh Fading).

The base simulation parameters are set as follows: basic observation window length $N_{b}=3$, confidence threshold $\beta \in \left\{0.02,0.06,0.10\right\}$, extension length $\Delta N\in \left\{2,4,6\right\}$, maximum iteration limit $I_{max}\in \left\{1,2,3\right\}$, normalized Doppler bandwidth $B_{d}T\in \left\{0.05,0.01,0.001\right\}$, and oversampling rate Q=8. For benchmarking, the traditional ML method described in [28] is adopted, aligning its observation window length with the total observation length $N=N_{b}+\Delta N$ of the proposed method to ensure fairness. The theoretical BER of coherent demodulation is also incorporated as a performance benchmark.

### 4.1. Parameter Optimization and Impact Analysis in AWGN Channels

Under the attenuation-dominated AWGN assumption, we first systematically evaluate the joint impacts of the three core configuration parameters: the confidence threshold β, the extension step size ΔN, and the maximum iteration limit $I_{max}$. This evaluation aims to identify the efficient operating point of the proposed generalized framework for short-range underwater EM links.

#### 4.1.1. Validation of the Confidence Metric Approximation

**Figure 5:**
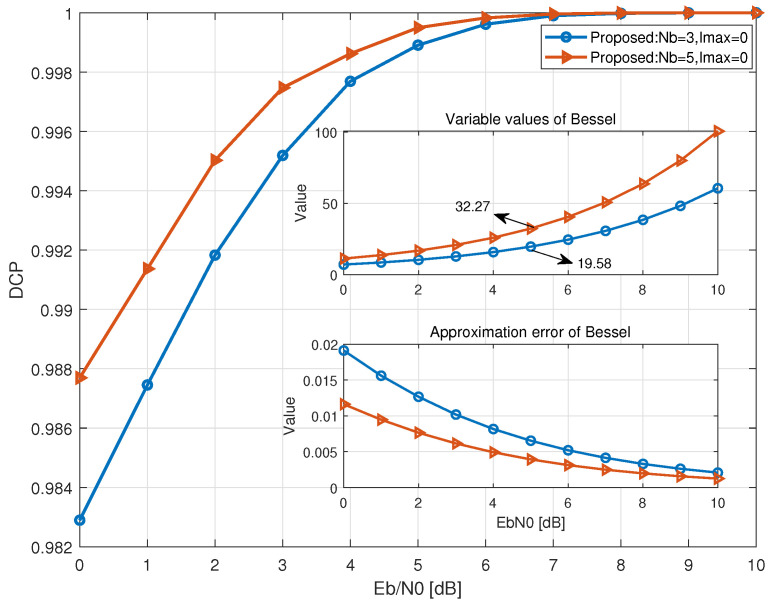
Validation of the confidence approximation: DCP, variablevalues and approximation error of Bessel function.

This subsection validates the approximation chain adopted in Section 3.2 for constructing the confidence metric, where the exact noncoherent likelihood with Bessel summation is simplified via max-dominance and the high-SNR approximation of $I_{0}\left(\cdot \right)$. To this end, we introduce the Decision Consistency Probability (DCP), defined as the ratio of the number of identical decisions to the total number of decisions. Figure 5 provides complementary evidence from the decision level and the function-approximation level.

As illustrated in Figure 5, a consistently high DCP is observed over the entire SNR range: The consistency remains 98% at $E_{b}/N_{0}=0$ dB and reaches 100% at $E_{b}/N_{0}=10$ dB, indicating that the approximation preserves the decision direction used in the confidence-driven control logic. Moreover, Figure 5 compares $N_{b}=3$ and $N_{b}=5$, showing that a larger observation window further increases the decision consistency (higher DCP), indicating enhanced agreement between the approximate and exact decisions as more observations are accumulated. The top insert depicts the Bessel argument $z=2\max_{k\in S_{i}}\Lambda \left(k,N\right)/N_{0}$ as a function of $E_{b}/N_{0}$. As $E_{b}/N_{0}$ increases, *z* grows monotonically, which moves the detector into the region where the exponential approximation of $I_{0}\left(z\right)$ is increasingly accurate. Consistently, the bottom insert compares the exact $I_{0}\left(z\right)$ with its exponential approximation and the approximation error remains small across the considered SNR range, staying within about 1% even at $E_{b}/N_{0}=0$ dB, and further decays rapidly as *z* increases.

Overall, Figure 5 confirms that the adopted approximation remains decision-consistent with the exact likelihood formulation and becomes progressively accurate as the operating SNR increases, supporting the use of $\alpha_{conf}$ for adaptive window control.

#### 4.1.2. Joint Impact Analysis of Confidence Threshold

**Figure 6:**
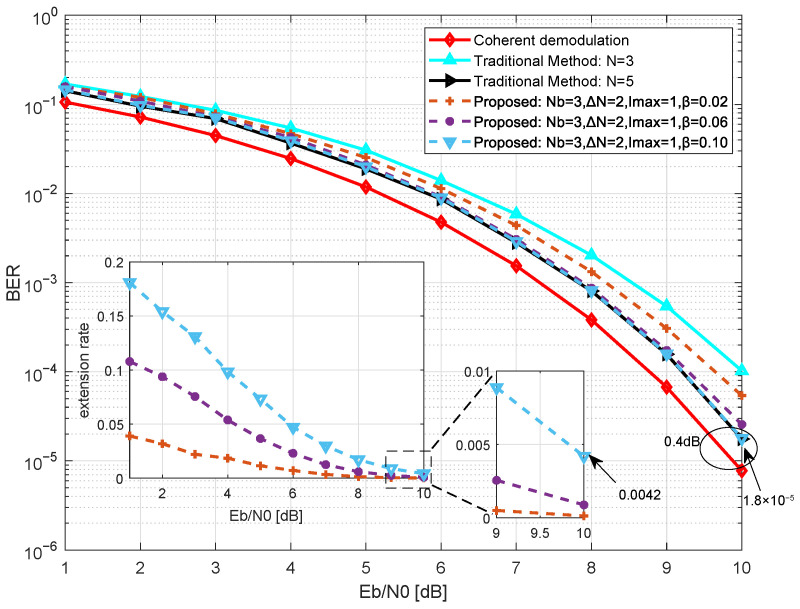
Simulation of BER and rate for the proposed method at different confidence thresholds.

We first analyze the impact of the confidence threshold on the demodulation performance and extension behavior of the proposed method in Figure 6. Simulations of BER and extension rate are conducted for different values of $\beta \in \left\{0.02,0.06,0.10\right\}$, with fixed $N_{b}=3$, ΔN=2, and $I_{max}=1$. The extension rate is defined as the ratio of symbols triggering the observation window extension to the total number of decision symbols.

As shown in Figure 6, by adjusting the confidence threshold, the proposed method flexibly enhances demodulation performance at the cost of a limited increase in extension rate. The main plot demonstrates that the BER performance of the proposed method improves as β increases. When β=0.10, the BER curve of the proposed method closely matches that of the traditional method (N=5), with only approximately 0.4 dB of performance gap relative to theoretical coherent demodulation. The inset shows that the extension rate γ increases monotonically with β. When β=0.10, the extension rate at $E_{b}/N_{0}=10$ dB is approximately 0.42%, indicating that the system relies solely on the base observation window for decision-making in 99.5% of cases. Simulation results indicate that at β=0.10, the proposed method maintains equivalent demodulation performance to traditional methods while keeping the extension rate extremely low. Therefore, β=0.10 can be considered an efficient operating point for the proposed method, significantly reducing computational complexity while maintaining demodulation performance.

#### 4.1.3. Joint Impact Analysis of Extension Length

We next investigate the impact of extension length on the upper limit of demodulation performance and resource efficiency of the proposed method in Figure 6. With $N_{b}=3$, $I_{max}=1$, and the efficient operating point β=0.10, the BER and AOL of the proposed method are simulated for different values of $\Delta N\in \left\{2,4,6\right\}$. Here, AOL is defined as the mean observation length actually used during symbol decision.

**Figure 7:**
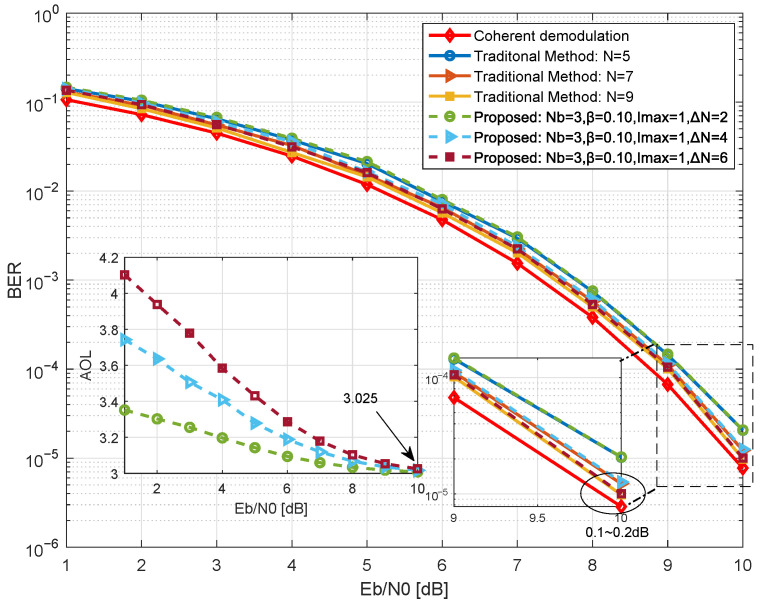
Simulation of BER and AOL for the proposed method at different extension lengths.

Figure 7 demonstrates that the extension length establishes a flexible performance ceiling for the system, while the average resource overhead of the proposed method remains close to that of the basic observation window. The main plot shows that as ΔN increases, the BER performance of the proposed method continues to improve, with all performance curves closely matching their corresponding traditional methods. When ΔN=6, the performance gap between the proposed method and theoretical coherent demodulation narrows to approximately 0.1 dB, indicating its potential to approach theoretical optimal performance. The inset illustrates that AOL increases with ΔN, but decreases with higher $E_{b}/N_{0}$. Under high $E_{b}/N_{0}$ conditions, AOL can be controlled within 3.1, approaching the base observation window length $N_{b}$.

#### 4.1.4. Joint Impact Analysis of Maximum Iteration Limit

**Figure 8:**
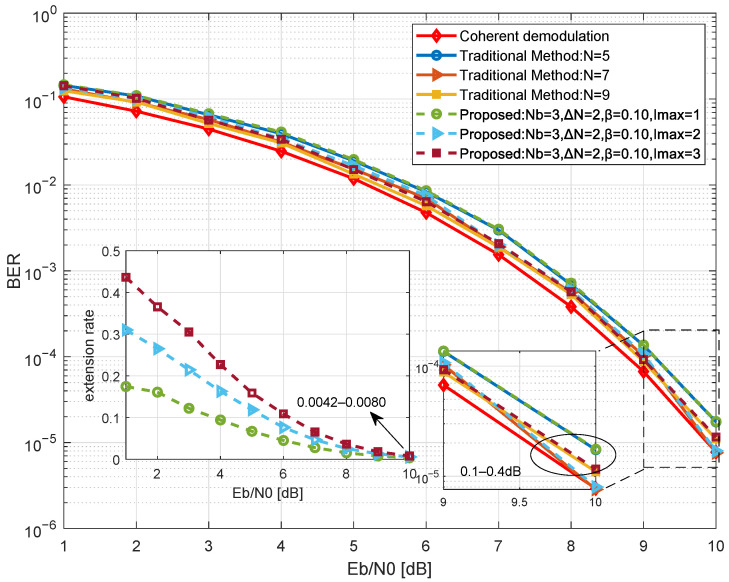
Simulation of BER and extension rate for the proposed method at different iteration limits.

Furthermore, we evaluate the impact of the maximum iteration limit $I_{max}$ on the demodulation performance and resource efficiency in Figure 8. With $N_{b}=3$, ΔN=2, and the efficient operating point β=0.10, simulations of BER and extension rate are conducted for different iteration limits $I_{max}\in \left\{1,2,3\right\}$.

As shown in Figure 8, multi-step iterative extensions ($I_{max}\geq 2$) effectively improve performance by continuously accumulating signal energy. The main plot demonstrates that increasing $I_{max}$ yields noticeable overall BER improvements to further combat severe noise. Meanwhile, the inset illustrates that although a larger $I_{max}$ introduces a higher extension rate at low $E_{b}/N_{0}$ to resolve high decision uncertainty, the rate for all configurations rapidly converges downward as $E_{b}/N_{0}$ increases. Therefore, configuring $I_{max}$ serves as an effective strategy to push the demodulation limits, trading a controllable and temporary increase in computational overhead for enhanced overall performance.

### 4.2. Robustness Evaluation and Parameter Impact Analysis in Rayleigh Fading Channels

Having established the operating point in the attenuation-dominated AWGN baseline, we further evaluate the proposed receiver under frequency-flat Rayleigh fading with a Jakes’ Doppler spectrum to emulate challenging multipath conditions. As discussed in Section 2.3, the noncoherent metric relies on a quasi-static assumption within the effective observation window; hence, the normalized Doppler bandwidth $B_{d}T$ and the effective window duration jointly determine the degree of metric mismatch and the resulting error floor. In this section, we first examine the impact of $B_{d}T$ and then use different base window lengths $N_{b}$ to reveal how excessive window growth becomes harmful in fast fading, thereby analyzing the performance of adaptive strategies under Rayleigh channels.

#### 4.2.1. Joint Impact Analysis of Normalized Doppler Bandwidth

The normalized Doppler bandwidth $B_{d}T$ characterizes the time-varying nature of the underwater multipath channel: A larger $B_{d}T$ indicates faster fading and a shorter channel coherence time $T_{c}$ [34]. Fixing the algorithm at the previously determined efficient operating point ($N_{b}=3,\Delta N=2,\beta =0.10$, and $I_{max}=1$), we simulate the BER performance and extension rate under $B_{d}T\in \left\{0.001,0.01,0.05\right\}$.

**Figure 9:**
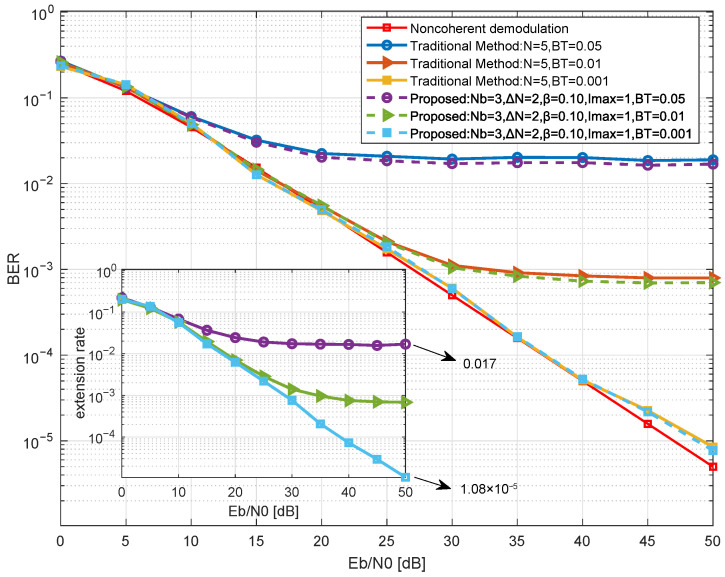
Simulation of BER and extension rate for the proposed method at different normalized Doppler bandwidth.

As illustrated in Figure 9, the BER curves degrade as $B_{d}T$ increases, and a more pronounced high-$E_{b}/N_{0}$ error floor is observed for larger $B_{d}T$. The inset further shows that the extension rate quickly drops with $E_{b}/N_{0}$ and remains extremely low in the high-$E_{b}/N_{0}$ regime. These results indicate that, under faster channel variations, the effective benefit of enlarging the observation window diminishes and the receiver performance becomes limited by time-variation-induced mismatch. This mismatch effect is inherent to noncoherent metrics developed for slowly varying phases and, thus, affects both the traditional fixed-window receiver and the proposed framework. Meanwhile, the consistently low extension rate at high $E_{b}/N_{0}$ suggests that the proposed strategy naturally avoids unnecessary window growth, which helps limit mismatch accumulation in time-varying fading.

#### 4.2.2. Joint Impact Analysis of Base Observation Length

In time-varying Rayleigh fading, increasing the observation window does not always improve performance, because a longer window may exceed the channel coherence time and aggravate metric mismatch. To illustrate this effect, we simulate traditional fixed-window receivers with N∈{5,7,9} under a representative slow-fading condition ($B_{d}T=0.001$), and compare them with the proposed adaptive method.

**Figure 10:**
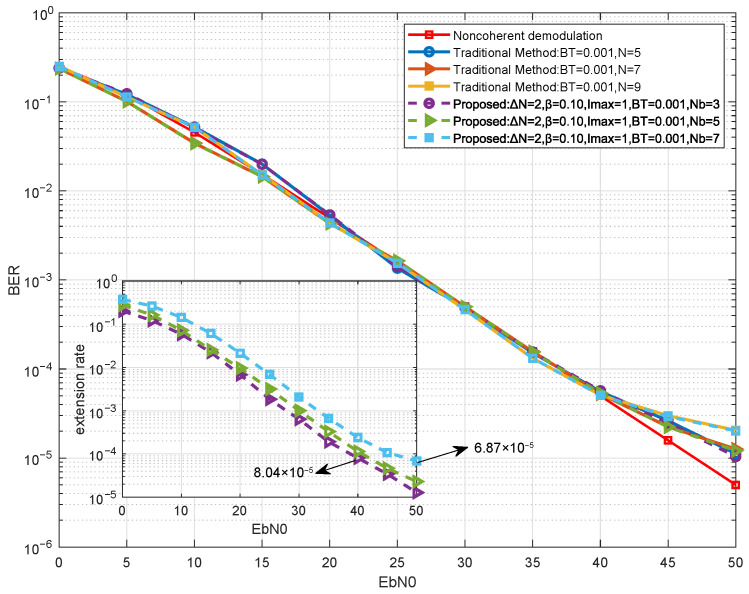
Simulation of BER and extension rate for the proposed method at different base observation lengths.

Figure 10 presents the BER performance of fixed-length traditional receivers with different observation windows under Rayleigh fading. At low $E_{b}/N_{0}$, different window lengths lead to nearly indistinguishable BER, indicating that performance is dominated by thermal noise. At high SNR, the longer-window configurations exhibit a mild degradation (about 4–5 dB at the considered BER level). This is because the quasi-static premise underlying the noncoherent metric is gradually violated over a longer window, so the resulting metric mismatch accumulates and manifests as an elevated error floor. The insert shows that the extension rate is extremely low at high $E_{b}/N_{0}$, implying that the proposed confidence-driven strategy rarely extends the window beyond the base length in this regime. Such behavior is consistent with the coherence-time constraint: Once the mismatch dominates, further extension provides little gain and may even be detrimental.

These results demonstrate that the robustness of the adaptive strategy stems not only from its ability to extend when necessary, but also from its inherent tendency to avoid extension when extension would be harmful. By operating predominantly with the short base window under favorable channel conditions, the method naturally respects the coherence time constraint, validating the confidence metric as an effective indicator of channel conditions in fading-dominated environments.

### 4.3. Computational Complexity Analysis

Building upon the optimal parameter configuration established in Section 4.1, we now quantitatively evaluate the computational efficiency of the proposed method. The analysis is divided into three parts: the computational execution operations compared against the established baseline, the strict quantification of the storage and memory access overheads, and latency bounds and hardware feasibility considerations [35,36,37,38].

#### 4.3.1. Computational Complexity and Baseline Selection

To ensure a rigorous evaluation of computational efficiency, the traditional fixed-window method with N=5 is selected as the primary baseline. This selection is predicated on achieving equivalent demodulation performance. As established in the previous section, the proposed adaptive method and the fixed N=5 receiver exhibit comparable bit error rates across the evaluated SNR range. While a fixed N=3 window would present lower absolute complexity, it incurs a severe BER penalty that renders it unsuitable for reliable underwater communication. Therefore, comparing against N=5 accurately reflects the computational savings our method provides while maintaining the required system reliability.

Table 1 presents a summary of the computational complexity of the proposed mechanism and the traditional method, in terms of number of operations, including complex correlation, sign flip, complex addition, and magnitude operation. To explicitly evaluate the engineering efficiency, the standard Floating-Point Operations metric is also included in the Table 1.

As shown in Table 1, the computational complexity of complex correlation is reduced from $O\left(N\cdot 2^{N}\right)$ in the traditional method to $O\left(2N\right)$ in the proposed mechanism. At the same time, the number of sign flip operations increases. This is because the proposed mechanism simplifies the traditional complex correlation operation into basic complex correlation and sign flip. Thus, the proposed method does not remove the exponential dependence on $2^{N}$ caused by exhaustive sequence search; instead, it substantially reduces the dominant correlation cost and decouples the oversampling factor *Q* from the exponential term.

By adopting the adaptive extension strategy, the computational complexity of the proposed method is further reduced. For clarity, we define the computation vector:

(22) $$v\left(N\right)=\left[C\left(N\right),F\left(N\right),A\left(N\right),M\left(N\right)\right].$$

Let the base observation window length be $N_{b}$, and the extension length be ΔN. Then the average computation vector per symbol can be expressed as

(23) $$\bar{v}\left(N_{b},\Delta N,\gamma \right)=\left(1-\gamma \right)\cdot v\left(N_{b}\right)+\gamma \cdot v\left(N_{b}+\Delta N\right).$$

Equation (23) indicates that the average complexity can be linearly adjusted via the extension rate γ.

Table 2 presents the computational complexity comparison results between the traditional method, the proposed mechanism and the proposed complete method, with typical parameters $N_{b}=3,\Delta N=2,\gamma =0.42\%$, and $I_{max}=1$.

As shown in Table 2, the number of complex correlation operations is reduced from 160 in the traditional method (N=5) to 10 in the proposed mechanism and further reduced by the proposed extension strategy. Due to the extremely low extension rate, the average computation load of the proposed complete method can be reduced to a level close to that of the traditional method (N=3). Compared to the traditional method (N=5), the proposed complete method reduces complex correlation operations by 96.2%, complex addition operations by 87.1% and magnitude operations by 74.7%.

Most importantly, the consolidated overall complexity metric demonstrates that the traditional N=5 method imposes a severe computational burden of approximately 10,272 FLOPs/bit. By applying the proposed adaptive extension strategy, the average arithmetic load is drastically compressed to approximately 428 FLOPs/bit. This profound 95.8% reduction in global FLOPs strictly validates the hardware efficiency of the proposed architecture, making it highly suitable for power-constrained underwater nodes without sacrificing demodulation reliability [39].

#### 4.3.2. Quantification of Storage and Memory Access Overheads

Similar to the computational arithmetic analysis, we explicitly quantify the spatial storage complexity and the dynamic memory access (read/write) operations based on the resource reuse mechanism. Table 3 presents a summary of the storage and memory access overheads for both the proposed mechanism and the traditional ML method.

As shown in Table 3, the traditional method computes correlations directly from received signals and bypasses intermediate static storage ($S_{T}\left(N\right)=0$). However, evaluating a single sequence fundamentally requires fetching all Q·N discrete signal samples. To traverse the entire search space of $2^{N}$ candidate sequences, the traditional method incurs an exponential number of memory read operations, scaling as $O(Q\cdot N\cdot 2^{N})$.

In contrast, the proposed mechanism structurally decouples the oversampling rate *Q* from the exponential search space $2^{N}$ [40]. The pre-computation phase requires exactly Q·N read operations to scan the received signal and 2N write operations to construct the base integral storage table. Subsequently, the likelihood composition requires exactly *N* pre-computed integral lookups for each sequence. Traversing all $2^{N}$ sequences, thus, necessitates exactly $N\cdot 2^{N}$ memory reads. Assuming a standard single-precision floating-point format where each complex variable occupies 8 bytes, the spatial complexity is strictly bounded to 16N bytes, representing a negligible constant O(N) storage requirement.

By adopting the adaptive extension strategy, the memory access overhead of the proposed method is dynamically optimized. For clarity, we define the memory overhead vector:

(24) m(N)=[S(N),R(N),W(N)],

Then the average memory overhead vector per symbol can be expressed as

(25) $$\bar{m}\left(N_{b},\Delta N,\gamma \right)=\left(1-\gamma \right)\cdot m\left(N_{b}\right)+\gamma \cdot m\left(N_{b}+\Delta N\right),$$

Equation (25) indicates that the average memory access complexity can also be linearly adjusted via the extension rate γ. To ensure a rigorous evaluation, the traditional method with N=5 is again selected as the primary baseline for performance parity.

Table 4 presents the quantitative comparison results between the traditional method, the proposed mechanism, and the proposed complete method, with typical parameters Q=8, $N_{b}=3$, ΔN=2, γ=0.42%, and $I_{max}=1$.

As shown in Table 4, the number of memory read operations is severely high in the traditional method (N=5), requiring 1280 distinct memory fetches per symbol decision. By utilizing the base integral table, the proposed mechanism (N=5) requires only 10 complex variables for static storage (amounting to merely 80 bytes) and reduces the memory reads to 200. Due to the extremely low extension rate (γ=0.42%), the average memory read operations of the proposed complete method remain tightly bounded at approximately 49.

Compared to the traditional method (N=5), the proposed complete method mathematically reduces memory read operations by 96.2%, fundamentally suppressing the exponential growth of memory bandwidth consumption at a negligible static storage cost. This hardware-agnostic analysis indicates that the proposed architecture achieves a profound net reduction in overall processing latency.

#### 4.3.3. Latency Bounds and Hardware Feasibility Considerations

To bridge the gap between theoretical algorithm design and practical engineering deployment, it is essential to evaluate the execution latency and hardware feasibility.

A common challenge in adaptive algorithms is the unpredictability of processing latency, which can disrupt real-time communication. In the proposed framework, the maximum processing latency is strictly deterministic and bounded by the predefined maximum iteration limit ($I_{max}$). The worst-case execution time occurs only when the window is extended to $N_{max}=N_{b}+I_{max}\cdot \Delta N$. Because $I_{max}$ is a rigid structural boundary configured prior to deployment, the system guarantees a deterministic upper bound on latency, ensuring reliable synchronization in real-time underwater links.

The proposed resource reuse mechanism effectively transforms exponential arithmetic operations into linear memory access operations. As quantified in Section 4.3.2, the absolute static memory cost is strictly bounded to tens of bytes, amounting to merely 80 bytes for the N=5 configuration. From a hardware architecture perspective, this negligible footprint is highly feasible for embedded implementation. It allows the lookup table to be entirely mapped into the Tightly Coupled Memory or distributed Block RAM of standard digital signal processors or Field Programmable Gate Arrays. This guarantees deterministic, single-cycle memory access, effectively avoiding the high latency and energy penalties associated with external DDR memory fetches. Consequently, the proposed method presents a highly feasible and energy-efficient solution for autonomous underwater nodes.

## 5. Conclusions

In this paper, we have proposed an innovative low-complexity ML noncoherent demodulation method for MSK systems in underwater electromagnetic communication. To balance complexity and performance, a two-stage optimization scheme has been constructed, incorporating a correlation resource reuse mechanism and an adaptive extension strategy. Compared with the traditional fixed-length ML noncoherent demodulator, the proposed method reduces complex correlation and complex addition operations by 96.2% and 87.1%, respectively, achieving a substantially lower average computational overhead while delivering a BER performance close to the ML benchmark. Furthermore, comprehensive simulations reveal that the proposed method demonstrates strong robustness not only in attenuation-dominated AWGN scenarios but also in time-varying Rayleigh fading channels. Under fading-induced phase and envelope variations, the proposed method maintains competitive demodulation performance and preserves its computational advantage.

## Figures and Tables

**Table 1:** Complexity comparison of proposed mechanism and traditional method.

Operation Type	Floating-Point Operations	Traditional Method	Proposed Mechanism
Complex Correlation	$6Q+2\left(Q-1\right)$	$C_{T}\left(N\right)=N\cdot 2^{N}$	$C\left(N\right)=2N$
Sign Flip	0	-	$F\left(N\right)=N\cdot 2^{N}$
Complex Addition	2	$A_{T}\left(N\right)=\left(N-1\right)\cdot 2^{N}$	$A\left(N\right)=\left(N-1\right)\cdot 2^{N}$
Magnitude Operation	3	$M_{T}\left(N\right)=2^{N}$	$M\left(N\right)=2^{N}$

**Table 2:** Complexity comparison results of proposed method and traditional method.

Operation Type	Traditional Method		Proposed Method		Reduction
	N=3	N=5	Mechanism	Complete	vs. Traditional
Complex Correlation	24	160	10	6	96.2%
Sign Flip	0	0	160	25	-
Complex Addition	16	128	128	16	87.1%
Magnitude Operation	8	32	32	8	74.7%
FLOPs/bit	1544	10,272	972	428	95.8%

**Table 3:** Storage and memory access comparison of proposed mechanism and traditional method.

Operation Type	Traditional Method	Proposed Mechanism
Static Storage	$S_{T}\left(N\right)=0$	$S\left(N\right)=2N$
Memory Read	$R_{T}\left(N\right)=Q\cdot N\cdot 2^{N}$	$R\left(N\right)=Q\cdot N+N\cdot 2^{N}$
Memory Write	$W_{T}\left(N\right)=0$	W(N)=2N

**Table 4:** Storage and memory access comparison results of proposed method and traditional method.

Operation Type	Traditional Method		Proposed Method		Reduction
	N=3	N=5	Mechanism	Complete	vs. Traditional
Static Storage	0	0	10	6	-
Memory Read	192	1280	200	49	96.2%
Memory Write	0	0	10	6	-

## Data Availability

Data are contained within the article.
